# Total Percutaneous Femoral Approach for Branched Custom-Made Device Endovascular Repair of Thoracoabdominal Aneurysm

**DOI:** 10.3400/avd.cr.20-00080

**Published:** 2020-09-25

**Authors:** Benjamin Dak Keung Leong, Feona Sibangun Joseph

**Affiliations:** 1 CardioVascular & Lung Centre, Gleneagles Hospital; 2 Unit of Vascular Surgery, Queen Elizabeth Hospital

**Keywords:** thoracoabdominal aneurysm, endovascular, steerable sheath

## Abstract

Thoracoabdominal aortic aneurysm (TAAA) is a challenging vascular condition to manage. Traditionally, open surgical repair has been the standard of treatment. Endovascular repair for TAAA has gained much popularity because it is a minimally invasive approach that results in better mortality and morbidity profiles. We report a case of TAAA successfully treated with a custom-made multi-branch device through a total femoral approach with the use of a steerable sheath for branch cannulation and deployment of bridging stents to targeted visceral vessels. This approach avoided complications related to upper extremity access, such as stroke, and allowed shorter operative time with better workstation ergonomics.

## Introduction

Thoracoabdominal aortic aneurysm (TAAA) remains a formidable disease to manage, particularly when there is invariable involvement of the celiac trunk, the superior mesenteric artery, renal arteries, or the artery of Adamkiewicz.

Traditionally, open repair has been the standard treatment for TAAA. It is, however, a major procedure. Recent advances in operative techniques and approaches with improved peri-operative care have resulted in better outcomes of the open surgery with a reported mortality rate of 7.5%–15.9% and morbidities, most notably a spinal cord ischemia (SCI) rate at around 2.6%–8.26% and post-operative renal failure at 5.7%–24.3%.^1–4)^

In recent years, total endovascular repair for TAAA has garnered popularity because of its minimal invasiveness. As compared to open repair, endovascular repair avoids extensive surgical dissection and hemodynamic manipulation leading to a lower mortality rate of 5.5% and a risk of SCI at 2.7%.^5)^ Nevertheless, in their series of TAAA repair, Greenberg et al. reported no difference between mortalities and SCI rates of open and endovascular approaches.^6)^ This, however, may only be representative for centers with large volumes and vast experience of open surgical TAAA repair.

We present a case report of successful treatment of TAAA through a total percutaneous femoral approach using a custom-made multi-branch device, hence, avoiding upper limb access altogether. To the best of our knowledge, this is the first intervention to be performed from total femoral approach in Malaysia.

## Case Report

**Figure 1:**
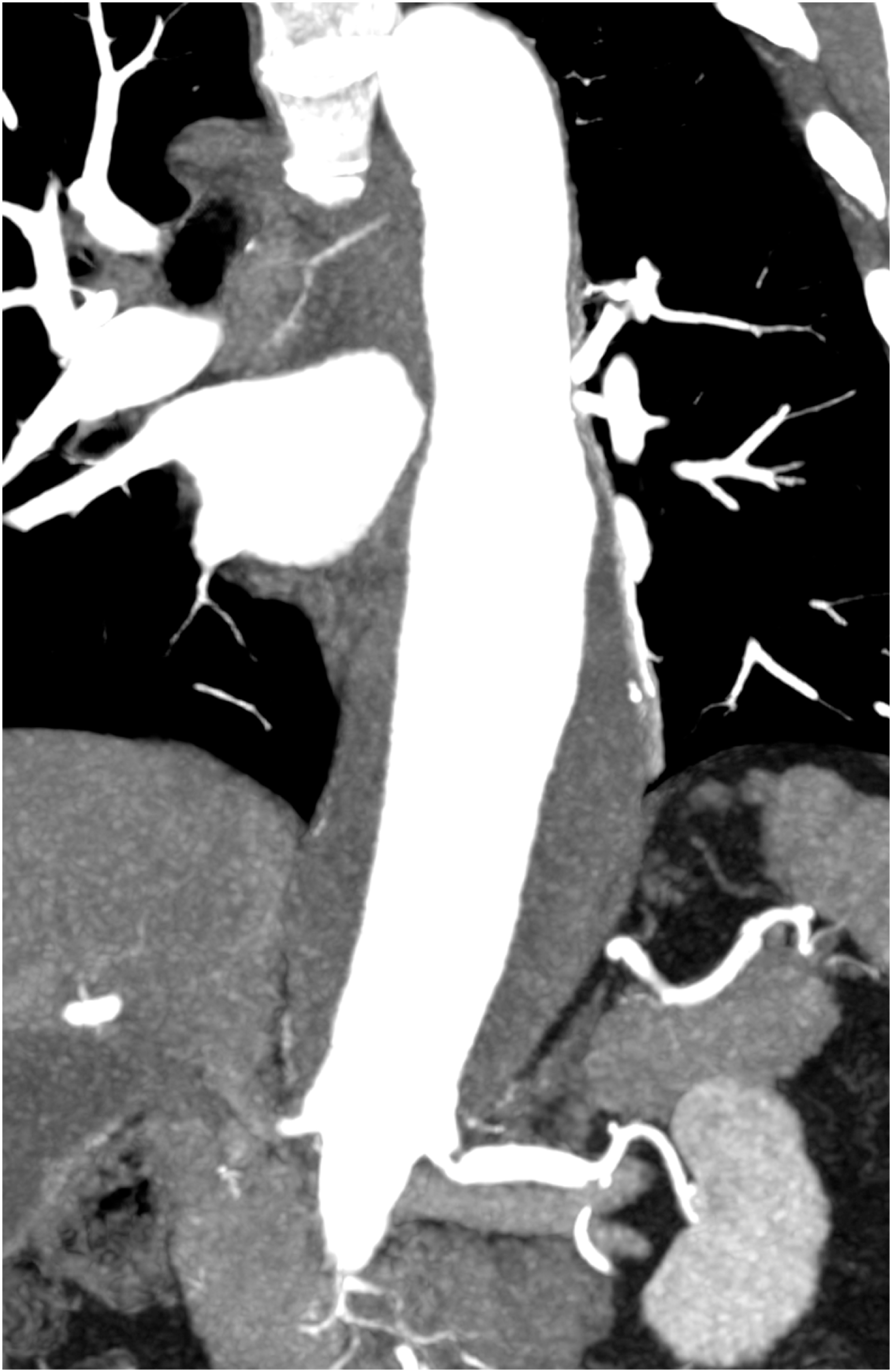
Fig. 1 Pre-operative computed tomography angiogram.

A 47-year-old man, with a history of Wegener’s granulomatosis and hypertension, presented with an acute onset of epigastric pain, which lasted for a few hours and resolved spontaneously. Peptic ulcer disease was ruled out after a normal gastro-duodenoscope study. A computed tomography (CT) scan revealed extent V (Safi) TAAA with a maximum diameter of 6.8 cm (**Fig. 1**). The celiac trunk was occluded. The superior mesenteric artery (SMA) had 50% stenosis at its origin with the prominent gastro-duodenal artery supplying the branches of the celiac trunk. Concurrently, there was bilateral renal artery stenosis, 70% at the left and 50% at the right. His creatinine was 122 µmol/L with an estimated glomerular filtration rate of 60 mL/min.

The patient had opted for endovascular repair for the management of TAAA after being counseled concerning options of open surgical and endovascular repair. A three-branched custom-made device (CMD) (Cook Medical, Brisbane, Australia) was planned for the procedure. Device manufacturing duration took 6 weeks. The procedure was performed under general anesthesia. A cerebrospinal drainage catheter was inserted for SCI prevention. Twelve grams of intravenous co-amoxyclav was administered as prophylaxis. Mean arterial pressure (MAP) was maintained above 90 mmHg throughout the procedure to prevent SCI. Access was obtained at both common femoral arteries through ultrasound-guided puncture followed by 5F sheath insertion on both sides. Two pieces of Perclose ProGlide (Abbott Vascular, Santa Clara, CA, USA) were deployed as a preclose approach at the right common femoral artery (CFA) followed by 8F sheath insertion. IV unfractionated heparin 3000 IU was delivered. Aortogram was performed through a calibrated pig-tail catheter from the left CFA access.

A proximal tapered thoracic endograft, Zenith Alpha, ZTA-PT-34-30-161 (Cook Medical, Bjaeverskov, Denmark) was deployed at the descending aorta with the distal end positioned 5 cm proximal to the left renal artery ostium. The CMD has proximal and distal diameters of 32 mm and 18 mm, respectively. The three branches were placed at the same level, measuring 8 mm, 6 mm and 6 mm in diameter with lengths of 18 mm, designed for the SMA and both renal arteries. Through a 20 F Flexor sheath delivery system from the right CFA, the CMD was advanced and properly oriented and deployed under fluoroscopy. The total stent covering length was around 25 cm. The delivery system was then removed with the Flexor sheath remaining in situ. Subsequently, a 16 F steerable sheath (22 mm deflected tip, 62 cm length), Heli-FX™ Guide (Medtronic, Santa Rosa, CA, USA), was delivered through the Flexor sheath.

**Figure 2:**
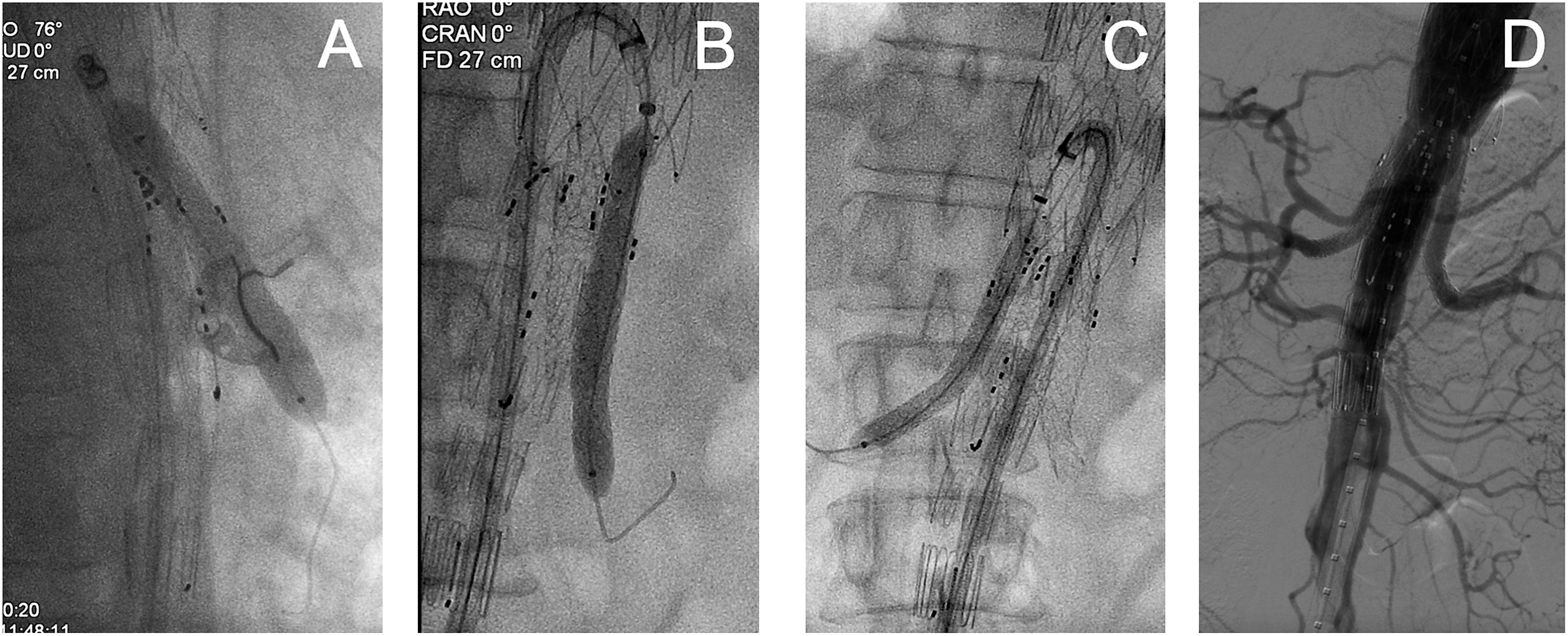
Fig. 2 The use of the steerable sheath for stent deployment into (**A**) the superior mesenteric artery, (**B**) the left renal artery and (**C**) the right renal artery. (**D**) Completion angiogram.

Cannulation of the SMA was performed with a 0.035 hydrophilic guide wire directed by the steerable sheath shaped into a bend of an approximately 180° turn. This was exchanged to a Rosen wire through a vertebral catheter. A 90 cm 7 F Rabee sheath (Cook Medical, Bloomington, IN, USA) was advanced to the ostium of the SMA for protection of the covered stent. Subsequently, a 10 mm×57 mm balloon-expandable covered stent, BeGraft (Bentley InnoMed, Hechingen, Germany), was positioned in place over the Rosen wire. The Rabee sheath was retracted to expose the stent before the stent was deployed. The same sequence was performed for the left and right renal arteries with 8 mm×57 mm and 6 mm×57 mm BeGraft, respectively (**Fig. 2**). Completion angiogram revealed that the SMA and the left and right renal arteries were well perfused with no major endoleak (**Fig. 2**). Operative time was 230 min, and fluoroscopy time 47.5 min with a radiation dose of 2,176 mGy.

**Figure 3:**
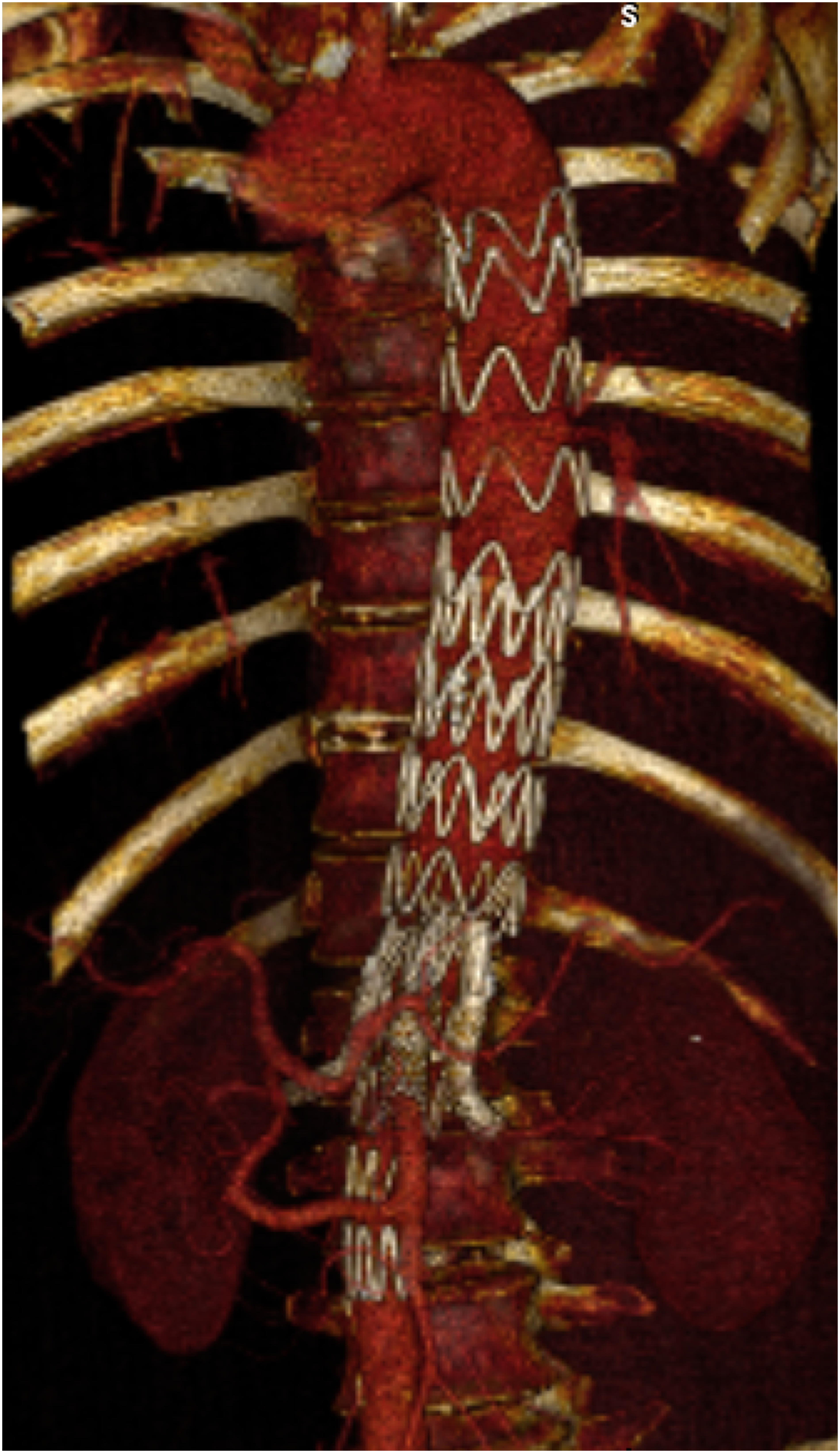
Fig. 3 Computed tomography angiogram 3 months post procedure.

Post-operatively, the patient was extubated and managed in intensive care unit. MAP was kept above 90 mmHg, and lumbar drainage was kept at 10 cm H_2_O with drainage not exceeding 10 mLs per hour. Spinal drain was clamped after 24 h and removed 24 h post-clamping. The patient had no immediate or delayed neurological deficiency throughout his recovery. However, he had developed features of post-implantation syndrome on post-operative day 2 with a temperature of 38°C and 21.7×10^9^/L raised white blood cells (WBCs). C-reactive protein (CRP) peaked on post-operative day 4 had a concentration of 236.3 mg/L. Fever persisted until post-operative day 5, but WBC and CRP showed a downtrend progress. The patient was discharged home on post-operative day 6 with paracetamol, aspirin and oral co-amoxyclav. CRP improved to 63.8 and 13.1, in the first and second weeks post discharge, respectively. A CT scan 3 months post procedure revealed no endoleak, and all three stented visceral vessels were well perfused (**Fig. 3**). His renal function has remained stable.

## Discussion

Endovascular repair for TAAA typically involves the use of branched devices, which can either be off the shelf, such as the Zenith t-Branch (Cook Medical, Bloomington, IN, USA), or custom-made. The choice largely depends on the urgency of intervention and anatomy of the aneurysm. The typical manufacturing time for a custom-made device is around 8–12 weeks, making it a less preferable option for urgent cases. Spanos et al. reported encouraging morality rate of 14% for urgent TAAA repair with t-Branch. However, repair of TAAA with anatomy outside instructions for use of t-Branch required a more technical effort, as well as operative and radiation time.^7)^ The first published systemic review on t-Branch reported an acceptable early mortality rate of 5.8% and a SCI rate of 12.2%. Up to 80% of cases in this review required a proximal thoracic extension graft to create a sealing zone in the diseased aorta, which resulted in longer operative time and a higher risk of SCI.^8)^

In branched endograft implantation of TAAA, the conventional access for cannulation of the branch, targeted visceral vessel and subsequent bridging stent is from the upper limb. This is achieved by either a percutaneous brachial artery access at the cubital fossa or a direct cut down of proximal brachial or infra-clavicular axillary artery. Access through the upper extremity will involve inevitable endovascular instrumentation over the origin of the vertebral artery. Particularly, right brachial or axillary artery access will involve direct instrumentation of the innominate artery and origin of the left common carotid artery and left subclavian artery. These maneuvers may increase the risk of aortic atheroma mobilization and cerebral embolization leading to stroke. Furthermore, upper limb access requires a wider operative workstation, often involving two separate teams positioned at upper and lower limbs of the patient. This is a less ergonomic working position with cross-over communication among teams and higher risk of radiation exposure for the team situated at the upper limb.^9)^

The use of the steerable sheath allows total percutaneous access from the femorals, thus avoiding access from the upper limb and possible complications described above. The steerable tip of the sheath supports appropriate angulation to be formed in relation to the origin of the branch and assists further cannulation of the target vessel. Additionally, the sheath provides a stable platform for the delivery and deployment of a bridging stent. Finally, through avoidance of access from above and assisted cannulation and bridging stent deployment, procedural time of such complex endovascular procedure is made shorter.^9)^ This is particularly beneficial for cases performed under an emergent setting.

## Conclusion

The endovascular management of TAAA is complex and often requires upper limb access for targeted branch management. As demonstrated in this case report, the use of steerable sheath facilitated a total femoral approach for cannulation and deployment of a bridging stent into targeted visceral vessels. This approach avoided access complications of the upper extremities, such as stroke, and allowed for a shorter operative time. In addition, it reduced the unnecessary risk of radiation by converging the operative team to a single work point.
